# Integrated analysis of expression profiles with meat quality traits in cattle

**DOI:** 10.1038/s41598-022-09998-w

**Published:** 2022-04-08

**Authors:** Yunxiao Li, Miaosen Yang, Angang Lou, Jinyan Yun, Chunyu Ren, Xiangchun Li, Guangjun Xia, Kichang Nam, Duhak Yoon, Haiguo Jin, Kangseok Seo, Xin Jin

**Affiliations:** 1 College of Life Science, Shandong University, Qingdao, China; 2 Engineering Research Center of North-East Cold Region Beef Cattle Science and Technology Innovation, Ministry of Education, Yanbian University, Yanji, China; 3 Department of Veterinary Medicine, College of Agriculture, Yanbian University, Yanji, China; 4 Department of Animal Science and Technology, College of Life Science and Natural Resources, Sunchon National University, Sunchon, South Korea; 5 Department of Chemistry, Northeast Electric Power University, Jilin, China; 6 Department of Animal Science, Kyungpook National University, Taegu, South Korea; 7 Branch of Animal Husbandry, Jilin Academy of Agricultural Sciences, Changchun, China; 8 College of Animal Science and Technology, Jilin Agricultural Science and Technology University, Jilin, China; 9 Animal Husbandry Bureau of Yanbian Autonomous Prefecture, Yanji, China

**Keywords:** Biological techniques, Biotechnology, Computational biology and bioinformatics, Genetics, Molecular biology, Zoology

## Abstract

MicroRNAs (miRNAs) play a vital role in improving meat quality by binding to messenger RNAs (mRNAs). We performed an integrated analysis of miRNA and mRNA expression profiling between bulls and steers based on the differences in meat quality traits. Fat and fatty acids are the major phenotypic indices of meat quality traits to estimate between-group variance. In the present study, 90 differentially expressed mRNAs (DEGs) and 18 differentially expressed miRNAs (DEMs) were identified. Eighty-three potential DEG targets and 18 DEMs were used to structure a negative interaction network, and 75 matching target genes were shown in this network. Twenty-six target genes were designated as intersection genes, screened from 18 DEMs, and overlapped with the DEGs. Seventeen of these genes enriched to 19 terms involved in lipid metabolism. Subsequently, 13 DEGs and nine DEMs were validated using quantitative real-time PCR, and seven critical genes were selected to explore the influence of fat and fatty acids through hub genes and predict functional association. A dual-luciferase reporter and Western blot assays confirmed a predicted miRNA target (bta-miR-409a and PLIN5). These findings provide substantial evidence for molecular genetic controls and interaction among genes in cattle.

## Introduction

Meat quality traits are complex genetic traits, which are crucial in meat quality improvement. The studies demonstrate that meat fat and fatty acid composition are influenced by genetic factors and are closely related to meat quality traits, including appearance, texture, flavor, juiciness, tenderness, and hardness^1–6^. It implies that fats and fatty acids may reflect complicated underlying genetic controls. These two factors may be considered major phenotypic indices for meat quality evaluation, aided by understanding its molecular genetic controls.

MicroRNAs (miRNAs) are noncoded small molecule RNAs widely found in eukaryotic organisms and approximately 19–24 nucleotides in length. MiRNAs are essential post-transcription regulatory factors and negatively modulate gene expression in animals at a post-transcriptional level through cleavage or translational inhibition^7^. RNAs use mature miRNAs to induce silencing complexes (RISCs) to modulate target gene messenger RNAs (mRNAs)^8^. When a miRNA is entirely complementary to a target mRNA, a miRISC directly cuts the target mRNA to reduce the level of gene expression. When a miRNA is paired incompletely with a target mRNA, the target gene translation is suppressible, and binding sites are mainly in the 3′ untranslated regions (UTR) of the target mRNA^9,10^. miRNA sequences are highly conserved in various species, from nematodes to cattle and humans, and thus considerably crucial to biology and developmental decisions^11–15^. The conserved Wat-son–Crick is pairing to the miRNA's 5′ region is called the miRNA "seed" ^16^. However, miRNA target sites from the multitude of 3ʹ-UTR segments exist and do not depend on seed sequence^17^. miRNAs also play an essential regulatory role in several biological processes^18–20^, such as cell proliferation^21^, differentiation^22^, and apoptosis^23^, as well as epigenetic changes^24,25^.

In cattle, miRNAs are considered to relate to embryonic development^26–28^, skeletal muscle function^29–31^, adipose^32–34^, and fat cell metabolism^35–37^. For example, miR-378 promotes the differentiation of bovine preadipocytes^38^; miR-2373-5p and miR-23b-3p are expressed highly in intramuscular fat^39^; miR-1 and miR-133 are muscle-specific and involved in the modulation of muscle proliferation^40^. Fluctuating hormones substantially affect gene activity and cause fat deposits in mammals^41–43^. In a castrated bull, the amount of testosterone in the body decreases or disappears, which reduces protein assimilation in the body, decreases muscle growth, and increases fat deposition. As the changing increases, the meat of steer exhibits increased fat content, improved taste, and enhanced tenderness^44–49^. In brief, hormonal changes between bull and steer cause the difference in meat quality and affect muscle and fat composition. Gene expression profiles help understand phenotypic differences, phenotypic effects, and underlying evolutionary mechanisms for individual genes^50^. However, differences in gene expression are especially worth exploring between bull and steer in the phenotypic traits of mammalian tissues.

The molecular mechanism of influences on meat quality traits relative to fat and fatty acid in cattle was explored in the present study. The experimental animals were divided into two groups based on the bull and steer. We assume a difference between the bull and steer selected to investigate the expression profiles of miRNAs and mRNAs. We conducted the integrated analyses of differentially expressed miRNAs (DEMs) and differentially expressed genes (DEGs) and identified the significant differential expression of miRNAs and mRNAs associated with meat quality in the *Longissimus dorsi* (LD). Subsequently, we performed the expression pattern and co-expression analyses on differentially expressed mRNAs. Moreover, an mRNA–miRNA interaction study was performed using computational prediction and expression relationship analysis. This primary focus is the in-depth analysis of critical genes and miRNAs related to fats and fatty acids to obtain a comprehensive view, reveal their molecular functions in lipid metabolism, and identify related regulatory pathways.

## Results

### Integrated analysis for DEMs and DEGs

A total of 18 DEMs and 90 DEGs were screened at rigorous threshold FC ≥ 2.0 and *P* ≤ 0.05 between bulls and steers, respectively (Supplementary Tables S6 and S7). The description of DEMs and mRNA was visualized using a volcano plot (Supplementary Fig. S1). Hierarchical clustering may have similar biological functions in the same cluster, as shown in Supplementary Figs. S2 and S3. The actual variations of expression are described in Supplementary Fig. S4. In this study, the target genes of miRNAs were predicted based on the transcript of Bos Taurus UMD 3.1.1 by using miRanda at 140 default threshold scores. A total of 5688 target genes for 18 DEMs were identified.

### Pathway, GO, and UniProt analysis for intersection genes

**Figure 1:**
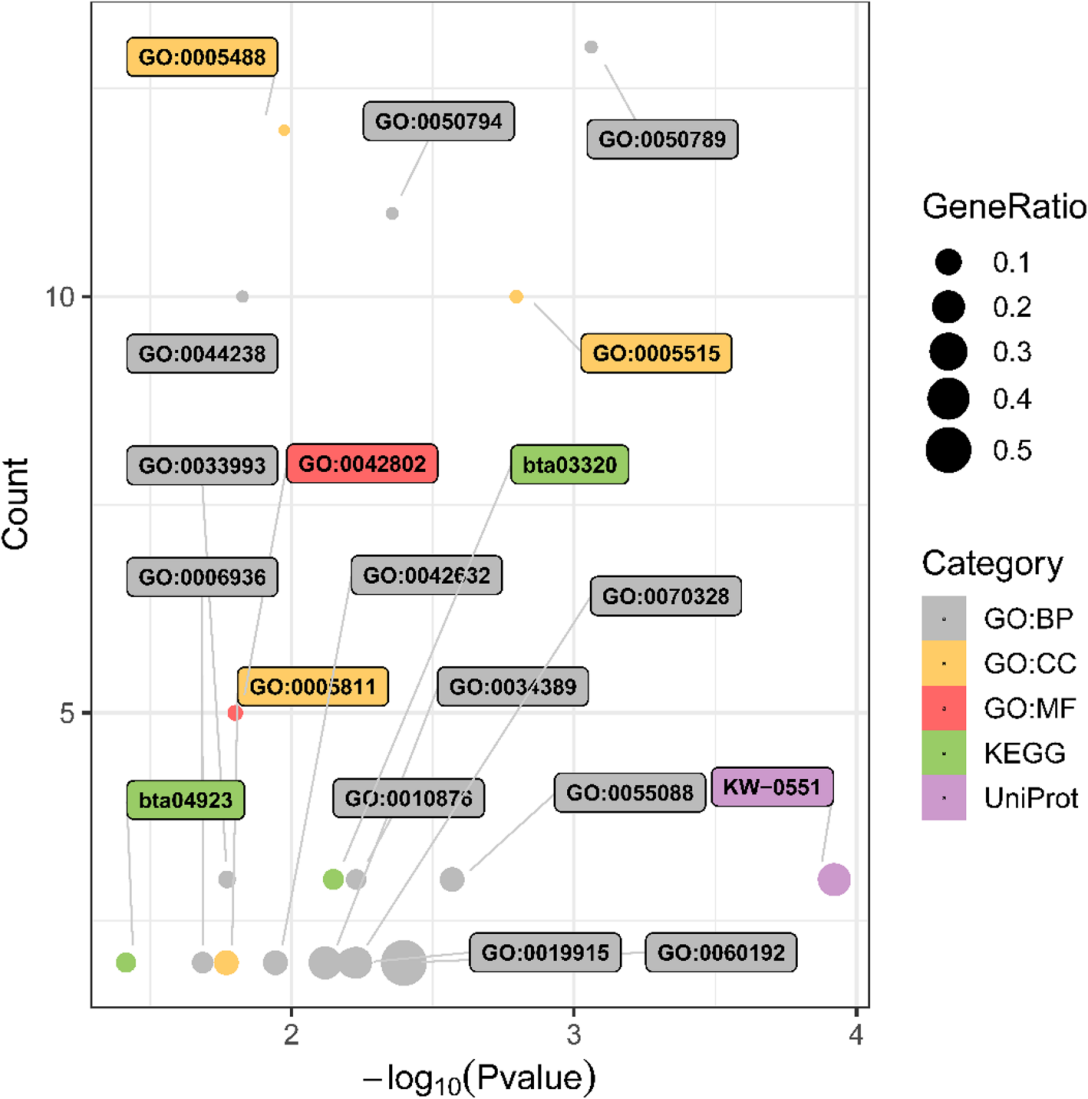
Significantly enriched GO, Pathway, and UniProt for intersection genes. *P* ≤ 0.05.

The significantly enriched GO, Pathway, and UniProt based on the 26 intersection genes were primarily involved in lipid metabolism, shown in Fig. 1. Some metabolic processes assessed were lipid droplets, identical protein binding, lipid homeostasis, the negative regulation of lipase activity, protein phosphorylation, lipid localization, lipid storage, triglyceride homeostasis, and response to lipid and muscle contraction. Exactly 17 genes of the 26 enriched in 19 terms were related to lipid metabolism (CIDEC, DGAT2, PLIN5, ABAT, ACVR1, ANGPTL4, DDIT4, HES1, ROCK2, S100A14, SCN4B, USP15, CSNK1A1, FABP4, ATG4A, CCNE2, and IRS1). Several genes were co-presented in multiple terms (Supplementary Fig. S5). Few genes enriched in the characterization of candidate canonical Pathway and UniProt. Significantly enriched pathways included the peroxisome proliferator-activated receptor (PPAR) signaling pathways (ANGPTL4, FABP4, and PLIN5) and the regulation of lipolysis in adipocytes (FABP4 and IRS1). Significantly enriched UniProt included the lipid droplet (CIDEC, DGAT2, and PLIN5).

### miRNA-mRNA interaction network analysis

**Figure 2:**
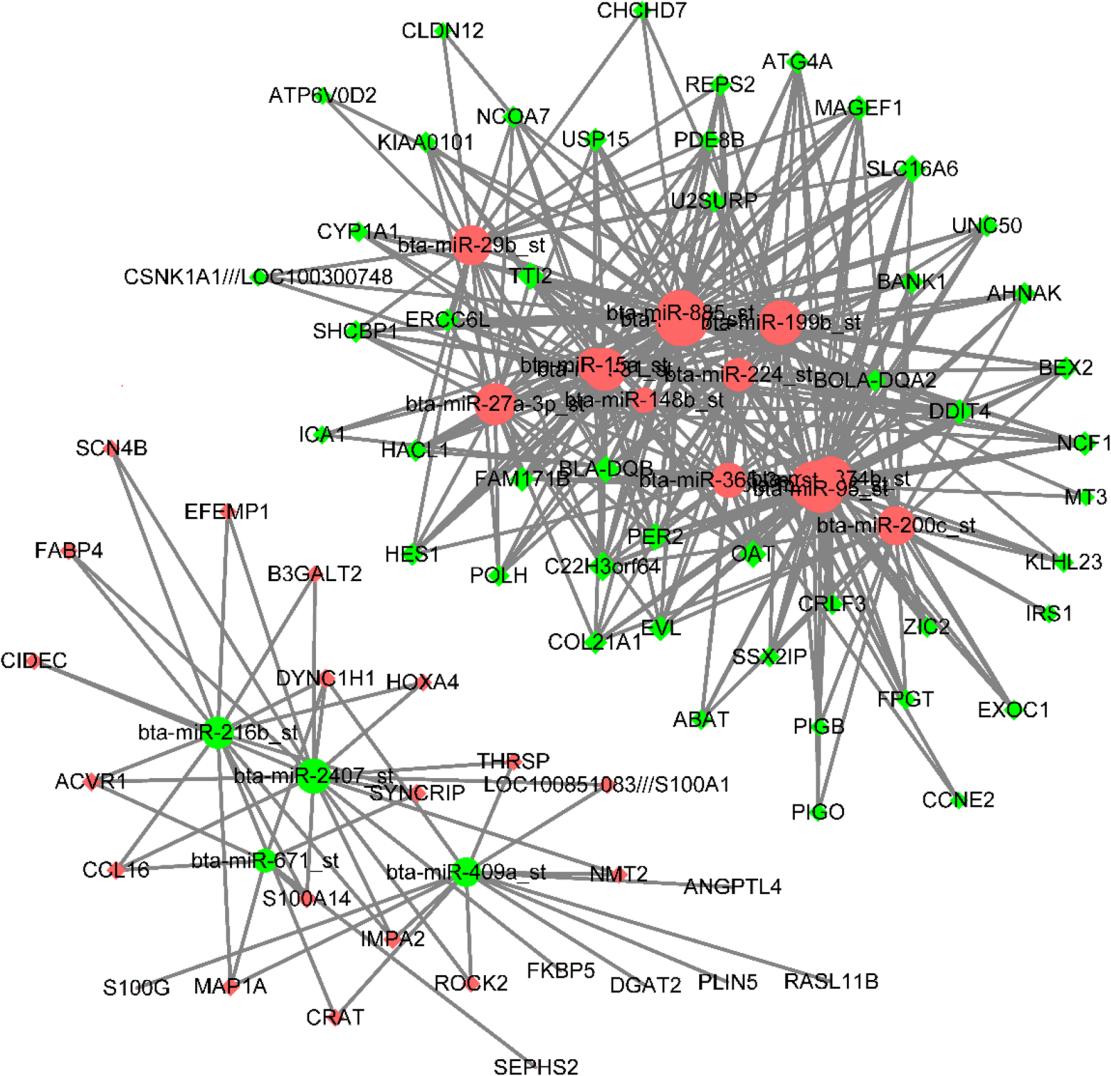
Interaction networks of miRNA and mRNA. Red and green indicate up- and down-regulation, respectively; round and diamond indicate regulated mRNA and miRNA, respectively; The significant round indicated that genes were regulated.

Using expression correlation and computation prediction (∣r∣ ≥ 0.8, *P* ≤ 0.05), we identified 83 potential mRNA targets for 18 miRNAs based on paired negative miRNA–mRNA expression profiling. The negative interaction network classed two subnetworks, including four miRNAs with regulated mRNAs (FABP4, CIDEC, ROCK2, THRSP, ANGPTL4, PLIN5, DGAT2, and CIDEC), and another included IRS1 with corresponding miRNAs (Fig. 2 and Supplementary Table S8).

A total of 75 matching target genes has been shown in the expression network. Among them, 26 target genes overlapped between DEGs and negative co-expression genes. These genes were an intersection gene for integration, as presented in Supplementary Fig. S6. In the 26 intersection genes, four down-regulated miRNAs were associated, and the relative expression of 12 genes increased, whereas the expression of 14 genes decreased in the bulls compared with steers. Most (14/26) genes associated with the 11 up-regulated miRNAs showed relatively increased abundances in steers, but the transcripts of 12 genes were down-regulated (Supplementary Table S9).

### Intragroup co-expression analysis

**Figure 3:**
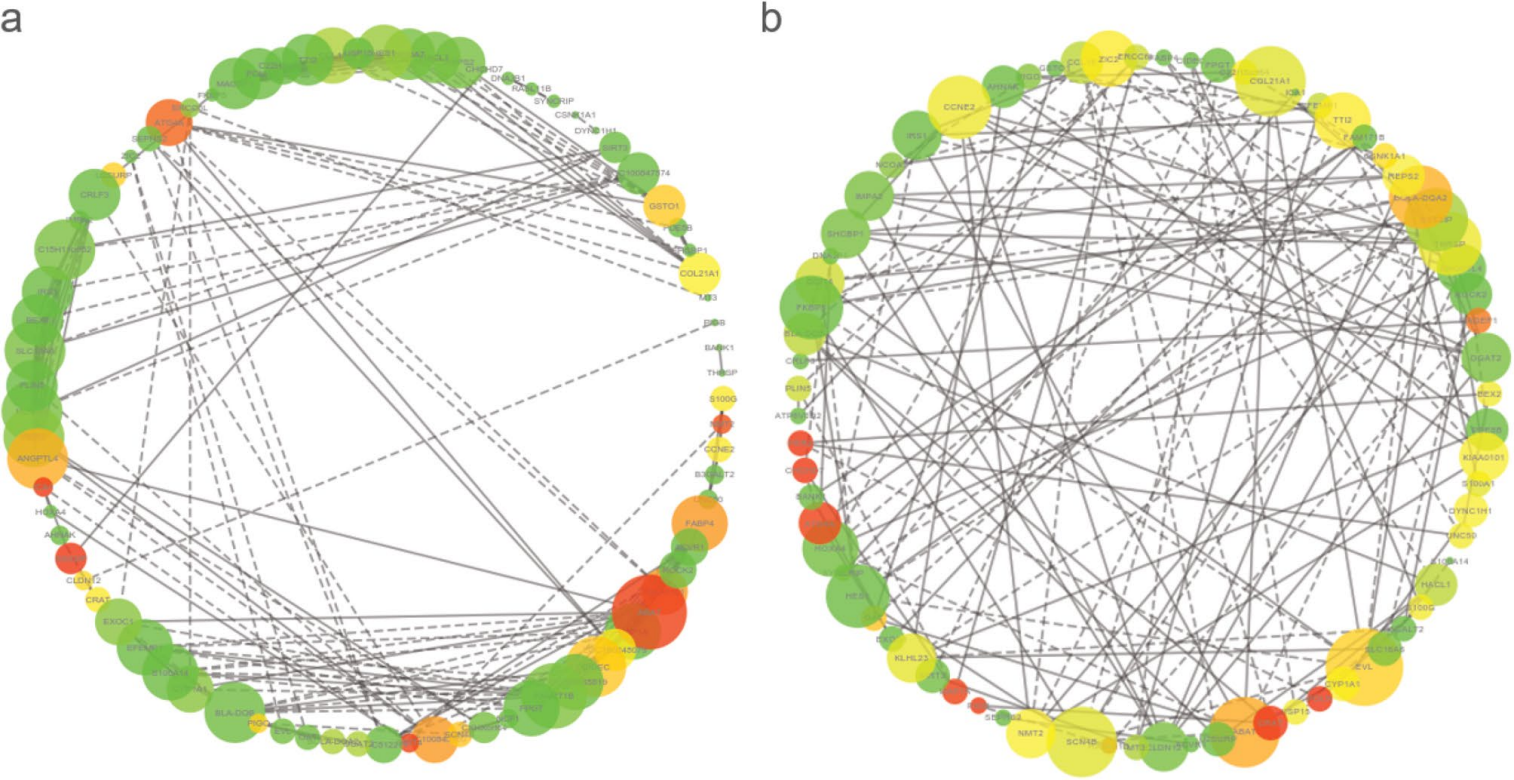
Co-expression analysis in bulls (**a**) and steers (**b**). Red indicates crucial nodes in the network, followed by orange and yellow; Green indicates a minimum contribution degree and low-value in the network; solid and dotted indicate pos- and neg-correlations, respectively.

The bull group (Fig. 3a) contains crucial co-expression genes, namely, ATG4A, ANGPTL4, FABP4, PIGO, ICA1, SSX21P, DDIT4 CIDEC, SCN4B, NMT2, GSTO1, COL21A1, CCNE2, and ABAT, were highly observed. The genes provide excellent contribution degrees and are indispensable for stable networks. Among them, CIDEC, FANBP4, ANGPTL4, ATG4A, and ABAT interacted with several genes. In the steer group (Fig. 3b), these crucial co-expression genes, namely, ATG4A, PER2, CHCHD7, MAP1A, PIGB, DDIT4, CIDEC, SCN4B, NMT2, CRAT, USP15, POLH, CYP1A1, EVL, DYNC1H1, KIAA0101, AGEF1, THRSP, COL21A1, CCNE2, PLIN5, ABAT, and DDIT4, were highly focused. These genes provided excellent contribution degrees and were indispensable for stable networks. CCNE2, DDIT4, CIDEC, EVL, ABAT, SCN4B, and ATG4A interacted with many genes.

### Hub genes and Co-expression predict the functional association

Four intersection genes, namely PLIN5, ANGPTL4, FABP4, and IRS1, were primarily observed in fat and fatty acid significantly enriched pathways. To further understand the intersection genes' function, we analyzed the top 10 hub genes using the cytoHubba calculation module of Cytoscape (Version 3.6.1). We observed six hub genes, namely DGAT2, FABP4, CIDEC, PLIN5, THRSP, and ANGPTL4, classed in a cluster and descending order (Supplementary Fig. S7).

**Figure 4:**
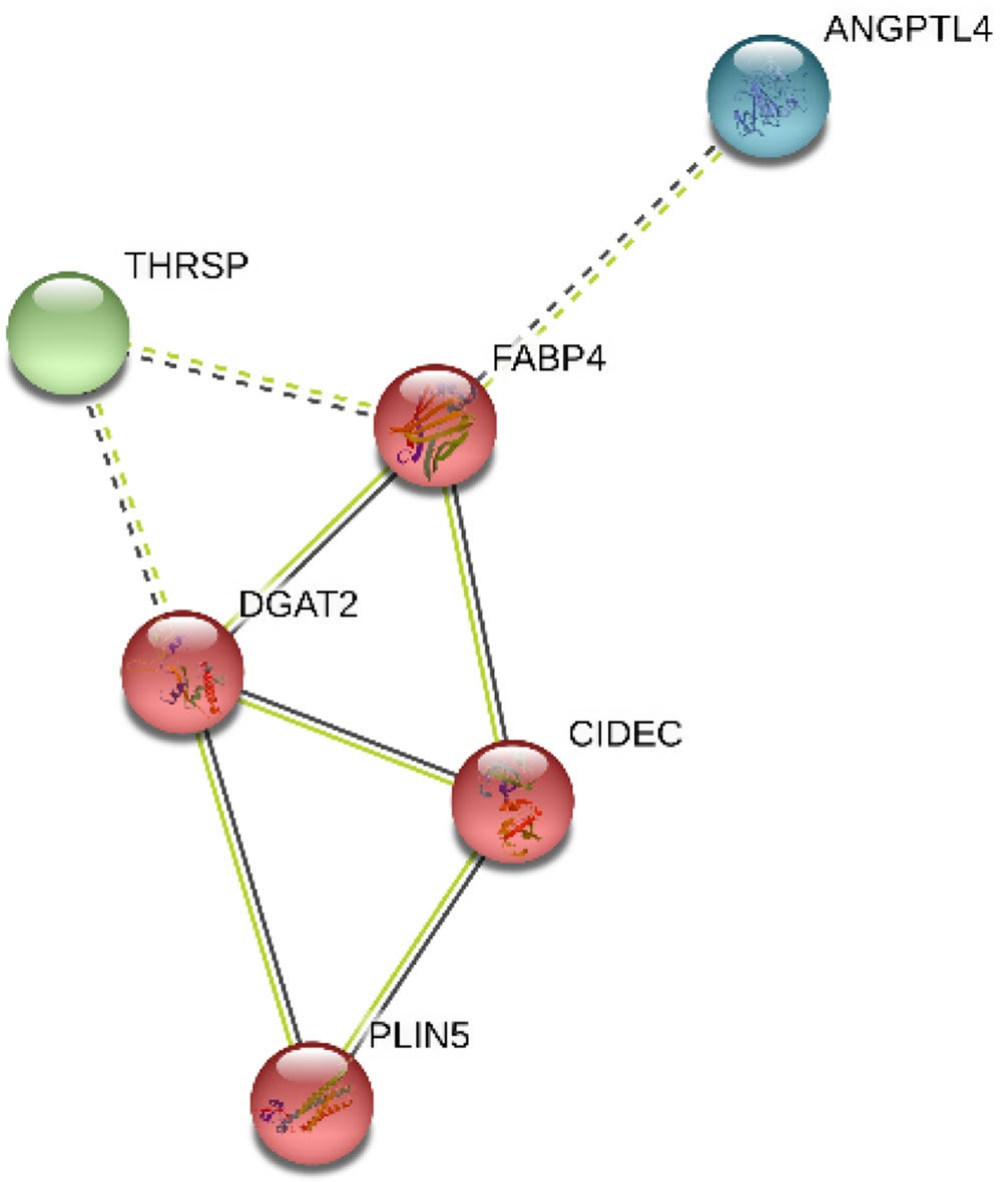
Overall expression data of interacting genes in the organism.

In the triangle matrices above, the intensity of the color indicates the level of confidence that two proteins are functionally associated, given the organism's overall expression data based on STRING (Version 11.0). Figure 4 shows the red genes representing strong confidence, their associations were specific and meaningful, and these genes jointly contributed to a shared function.

**Figure 5:**
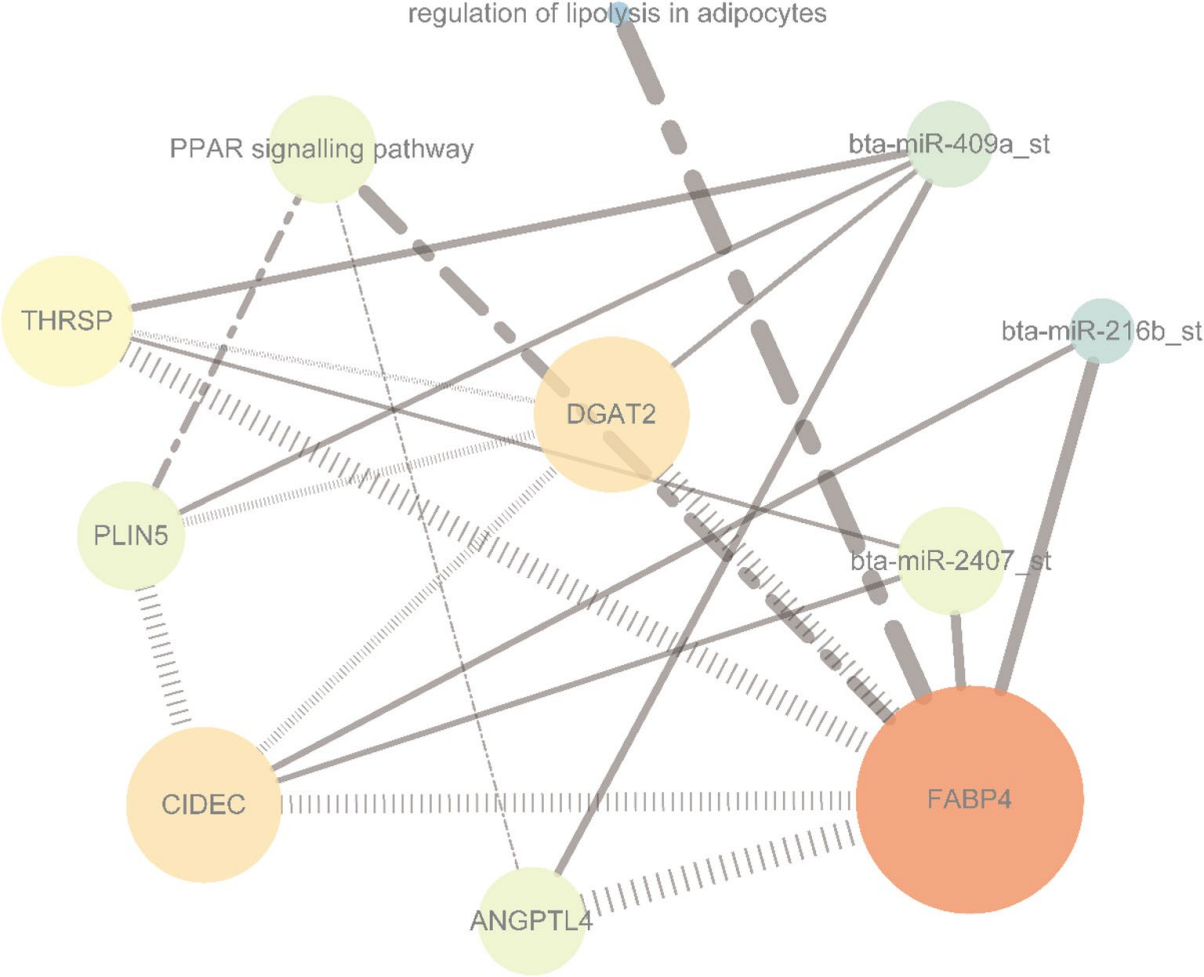
Interaction network for six critical genes with corresponding DEMs. Round and square indicate up- and down-regulation, respectively; Diamond and dash-dot indicate they involved pathways and enriched genes in the pathways, respectively; Vertical slash indicates the functional association between genes; Solid indicates a regulated relationship between gene and miRNA; Orange indicates the most strong closeness centrality, followed by yellow, green, and blue; Size is influenced by radiality; Line thickness is influenced by edge betweenness.

**Figure 6:**
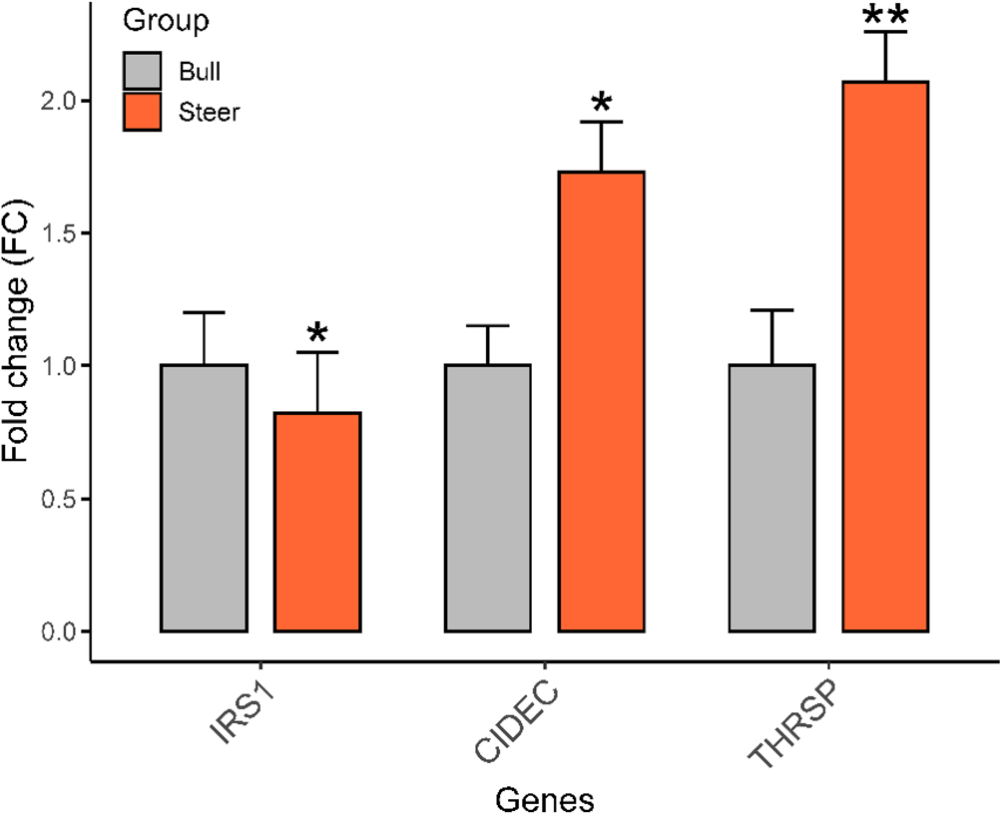
Validation of three critical genes by qRT-PCR, *P < 0.05, **P < 0.01.

A total of seven genes were critical genes, namely, PLIN5, DGAT2, ANGPTL4, CIDEC, THRSP, FABP4, and IRS1. Although DGAT2, CIDEC, and THRSP were not found in the pathways, they presented in the interaction network, intragroup co-expression, and UniProt. To visualize and understand the interaction between them and their corresponding DEMs, we constructed an interaction network between miRNAs and genes in Fig. 5, and the involved genes included in IRSI were further validated by qRT-PCR (Fig. 6).

**Figure 7:**
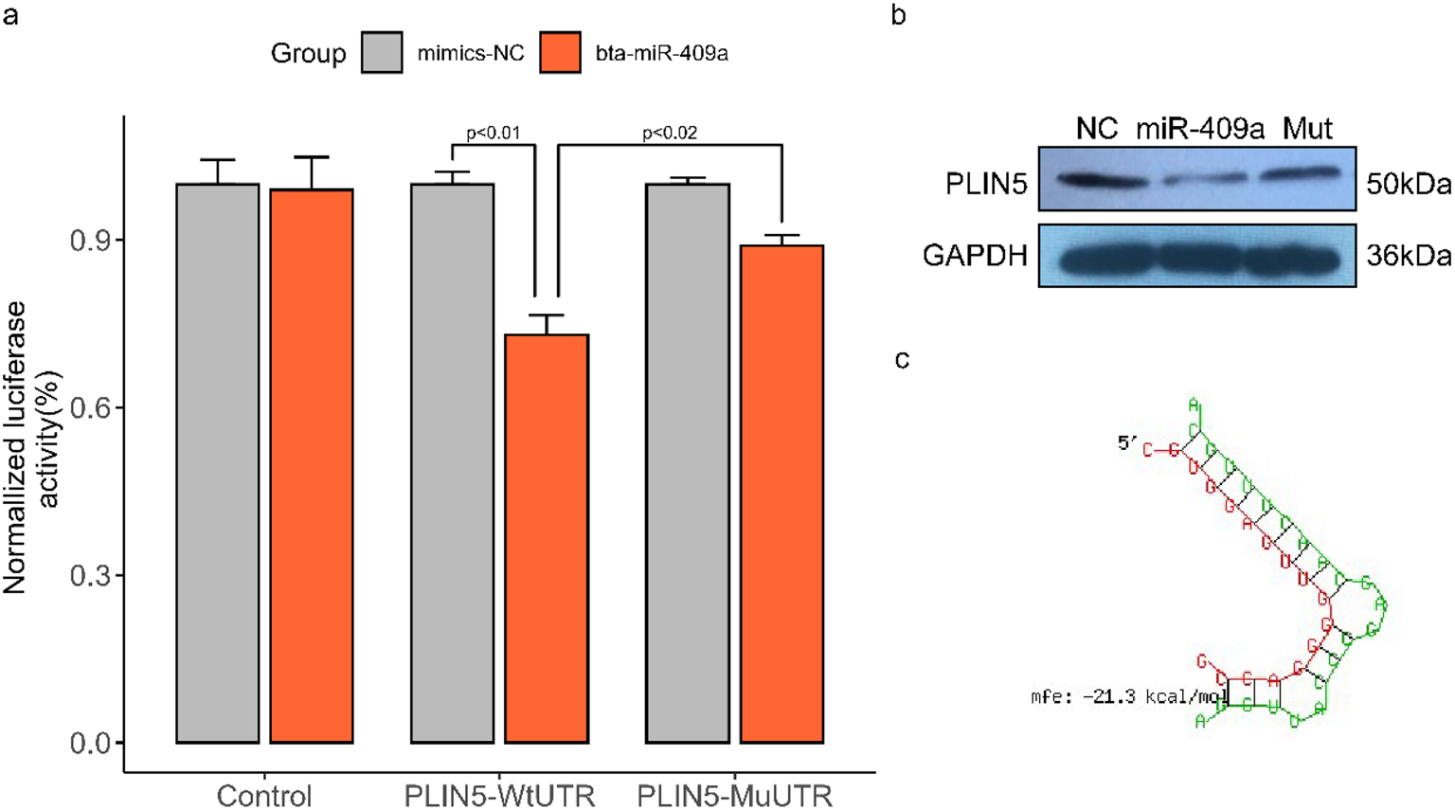
Validation of miRNA endogenous targets via dual-luciferase reporter and Western blot assay. (**a**) Showed the activity of PLIN5 and bta-miR-409a by binding assay. (**b**) Presented the validating of PLIN5 as a target of bta-miR-209a at the protein level using western blot. Three tracks were captured from the original full-length membrane image and were presented in the same order consistent with (**a**). (**c**) is nucleotide sequences of binding sites located in the 3′ UTR of PLIN5. The sample derives from the same experiment, and that blots were processed in parallel. The party of the full-length membrane image is presented in Supplementary Fig. S8.

According to the predicted binding sites on the miRNA regulation of target genes, the transcripts of the PLIN5 gene may be targets consistent with miRNAs. This finding was explored by the co-transfection of luciferase reporter vectors containing the wild-type or mutant 3′ UTR of genes. As shown in Fig. 7, the luciferase activities of the wild-type gene reporter co-transfected with the miRNA mimic were reduced significantly compared with that co-transfected with the negative control mimic or in mutated-type reporters co-transfected with the miRNA mimic. Compared with the negative control, the mRNA level significantly decreased in bovine adipocytes at 48 h after transfection with the miRNA mimic.

## Discussion

### MiRNA, fat and fatty acid in mammals

MiRNAs are small RNAs that regulate gene expression post-transcriptionally by repressing the translation and promoting the degradation of target mRNAs^51^. A miRNA typically has hundreds of evolutionarily conserved target sites, yet only very few predicted targets are down‐regulated more than twofold in miRNA transfection experiments^51–53^. Thus identifying target mRNAs is crucial to the understanding of the biological functions of miRNAs. At present, microarray-based techniques have been used in identifying mRNA–miRNA interactions, and negative expression correlations between miRNAs and their target mRNAs can be determined^54,55^. This strategy can significantly decrease the false-positive rate for identifying the miRNA with mRNA targets and improve laborious and time-consuming validation processes^56^. To date, the expression of miRNAs related to fat and fatty acid is still rarely reported between steers and bulls^58^.

The fat and fatty acid showed a significant difference between bulls and steers (Supplementary Tables S1, S2). It provides strong evidence to support the data of the experiment group that can be used for further analysis. The DEMs quantified, bta-miR-365-3p, bta-miR-122, bta-miR-200c, bta-miR-374b, bta-miR-15a, and bta-miR-671 were up-regulated in the LD of steers, and RT-qPCR confirmed the results. These significantly expressed miRNAs also were frequently reported in mammals. For example, A study identifies that activin A receptor type I (ACVR1) could be a direct target of miR-365-3p and further elucidate that miR-365-3p inhibits proliferation but promotes differentiation of primary bovine myoblasts by targeting the activin A receptor type I^59^. In human overlapping QTL for obesity, miR-365-3p is recognized in brown adipose tissue^60^. MiR-122 represents about 70% of the total miRNA in the liver, and its down- or up-regulation can modify fatty acids and cholesterol metabolism^61,62^. MiR-122 also associates lipid metabolism and adipocyte differentiation in cattle^63–65^, regulated PPAR-γ signaling and adipocyte differentiation in vitro and human adipose tissue^66^. MiR-200c is mentioned high expression in the mammary gland and present in milk whey^67,68^. MiR-374b has higher expression in the *Longissimus lumborum* muscle of grazing cattle than grain-fed Japanese Black cattle, as analyzed by qRT-PCR^69^. MiR-15a is reported more intensively in bovine mastitis. The studies indicate that miR-15a constitutes potential miRNA-mRNA regulatory pairs with target gene (IRAK2) for use as biomarkers to predict a mastitis response or indirectly affects the expression of CD163 gene in *E. coli*-infected mastitis cows^70–72^. MiR-671 and miR-7 largely interact with circRNA CDR1 antisense RNA (CDR1as; also known as ciRS-7)^73,74^. MiR-671 is up-regulated in the tissues from cows with mastitis^75^ and highly expressed in intermuscular fat^65^. MiR-7 and miR-15a may play a role in the variation of residual feed intake^76^.

In summary, these findings indicated that they might play essential or potential regulatory roles in lipid mechanisms in cattle. In comparison, changes in the three lipid-related DEMs (bta-miR-216b, bta-miR-409a, and bta-miR-2407) identified by miRNA profile and confirmed by qRT-PCR were down-regulated in the LD of steers. MiR-216b inhibits heat stress-induced cell apoptosis by targeting Fas in bovine mammary epithelial cells and modulates cell proliferation during early embryo development via K-RAS in cattle^77,78^. MiR-409a is up-regulated in bovine follicular atresia relative to healthy follicles^79^ and significantly down-regulated in preovulatory dominant follicles^80^. However, miR-409a related to lipid metabolism was down-regulated in the LD of steers in this study. An extensive literature search in PubMed and other databases did not retrieve any published results for miR-2407 in cattle.

### Critical genes, fat and fatty acids in mammals

Seven critical genes were identified, four of which are involved in lipid metabolism pathways. The roles of ANGPTL4, FABP4, THRSP, and DGAT2 were elucidated. DGAT2and PLIN5 enriched in lipid droplet (GO:0005811), lipid storage (GO:0019915), and lipid droplet (KW-0551). ANGPTL4, FABP4, and PLIN5 were significant in PPAR signaling pathway (bta03320). FABP4 and IRS1 appeared in the regulation of lipolysis in the adipocytes pathway (bta04923). Bovine ANGPTL4 is a critical enzyme in lipolysis that stimulates the oxidation of fatty acids and inhibits fat accumulation by inhibiting lipoprotein lipase activity and is high in subcutaneous adipose tissue^81^. DGAT2 gene acts in the deposition of saturated fat in the adipose tissue^82^ and is identified as a functional candidate gene affecting milk production, especially for fat content in milk^83^. THRSP is expressed in mature adipocytes rather than in the early stages of adipogenesis, demonstrate that an increased expression of THRSP in *Longissimus dorsi* is a consequence of but not the reason for a higher number of intramuscular adipocytes in cattle with enhanced intramuscular fat deposition^84^. FABP4 is identified as a lipid transport protein in adipocytes and belongs to the FABP family, is a relevant candidate gene for beef quality. It correlates with intramuscular fat content^85^, fatty acid compositions^86,87^, and inhibits the expression by miR-130a/b in adipocyte differentiation ^88^.

Among the other rarely reported critical genes, CIDEC was mentioned maybe a potential earlier predictor of the marbling potential of differential intramuscular fat^89^ and lipid droplet^90^. IRS1 may be related to yak milk protein synthesis during the lactation cycle^91^. PLIN5 involved the regulation of lipid and was widely reported to relate to maintaining the balance between lipogenesis and lipolysis in humans^92^. A study suggested that PLIN5 may play a crucial role in regulating lipids deposition as code for proteins coating intracellular lipid droplets surfaces in pigs, and further pointed out that variations in the PLIN5 sequence may be linked to Hormone-sensitive lipase (LIPE) gene expression through a still poorly known regulative molecular process^93^. However, the knowledge of the PLIN5 gene in the lipid metabolism of cattle is still incomplete^94^. Given that they displayed significant differential expression except for critical genes, these genes must be further studied. Additional genes identified here include ATG4A, CCNE2, CSNK1A1, ABAT, HES1, and ROCK2. The effect of these genes on lipid metabolism or fat accumulation is not apparent; thus, these genes require further study in bovine fat and fatty acids.

In the interaction network of miRNA–mRNA, three DEMs, namely, bta-miR-2407, bta-miR-409a, and bta-miR-216b, up-regulated the critical genes. They may be strong candidates for regulating fats and fatty acids because independent qRT-PCRs between bulls and steers differentially express these three DEMs. Under the influence of bta-miR-409a, the activity of the wild-type of PLIN5 decreased in the dual-luciferase reporter assay. After the 3′ UTRs of genes were mutated artificially, the mutated-type activity was higher than that of the wild-type. This finding indicated that mutations were essential for miRNAs binding. Therefore, we supposed that PLIN5 is perhaps involved in lipid transport and storage protein in adipocytes or has aroused binding to lipid droplets, regulates their enlargement, and promotes incorporating endogenously synthesized fatty acids into triglycerides by down-regulating bta-miR-409a in cattle^95–97^. In brief, FABP4 and DGAT2 may play a central role in regulates lipid adipocytes and fatty acids. CIDEC and PLIN5 may be closely associated with lipid droplets and regulate their enlargement, highlighted in intragroup co-expression of steers and UniPort. In addition, PLIN5 is probably involved in lipid droplet homeostasis by regulating the storage of fatty acids in the PPAR signaling pathway (Fig. 1 and Supplementary Fig. S5)^95^. A close interaction relationship may exist among the four genes from hub genes, co-expression predicts, and intragroup co-expression. However, because part of anti-gene antibodies suited for bovine were lacking, changes in the level of some protein expression were not measured. Therefore, these actual results showed in Fig. 7.

In conclusion, differences between steers and bulls in the mRNAs and miRNAs related to fats and fatty acids were demonstrated. The integrated analysis of DEMs and DEGs suggests that three miRNAs (bta-miR-409a, bta-miR-2407, and bta-miR-216b) and seven critical genes (FABP4, IRS1, ANGPTL4, THRSP, CIDEC, DGAT2, and PLIN5) are strong candidate miRNAs and genes involved in regulating the fat and fatty acids in cattle. Potential miRNAs (bta-miR-365-3p, bta-miR-122, bta-miR-200c, bta-miR-374b, bta-miR-15a and bta-miR-671,) and genes (ATG4A, CCNE2, CSNK1A1, ABAT, HES1, and ROCK2) related to lipid metabolism were also identified. The study results indicate that bta-miR-409a that interacted with PLIN5 may play an important role in lipid droplets and fatty acid composition. The challenge for future studies is to identify the other relevant targets of miRNAs and determine the interaction among genes and contribute to the regulation of lipid droplets and fatty acid compositions.

## Materials and methods

### Ethics declarations

Following the protocols approved by Jilin Province, P. R. China for Biological Studies Animal Care and Use Committee, all experimental procedures were approved by the Animal Ethics Committee of Yanbian University and conducted in strict compliance with the recommendations (approval number: 2018062815-4). All efforts were made to minimize animal suffering.

### Animals

Six-month-old Yanbian cattle were selected from a Jixing farm in Jilin province. After 30 days of fattening, 60 bulls were selected for castration. All experimental cattle in the same feeding conditions (Supplementary Table S10) were fattened to slaughter under the commercial standard at 36 months. The carcass was stored at 0–4 °C and bio-acid treated for three days. The 60 bull and 60 steer samples were obtained from LD between the 12th and 13th right ribs and stored at − 20 °C for meat quality determination. Three bull and three steer fresh tissue samples were randomly selected and cut into small 0.3 cm^3^ blocks, immediately placed in frozen tubes, and stored in liquid nitrogen to further detect miRNA and mRNA expression profiling.

### RNA extraction

Total RNA was isolated from frozen tissue samples (*n* = 6) using mirVana™ RNA Isolation Kit (Applied Biosystem, Invitrogen) according to the manufacturer's instructions and treated with RNase-free DNase I to remove genomic DNA contamination. RNA integrity (RIN ≥ 7, 28S/18S ≥ 0.7) was assessed using an Agilent 2100 Bioanalyzer Lab-on-chip system (Agilent Technologies, USA). The same samples were used in all experiments.

### Fat and fatty acids

**Figure 8:**
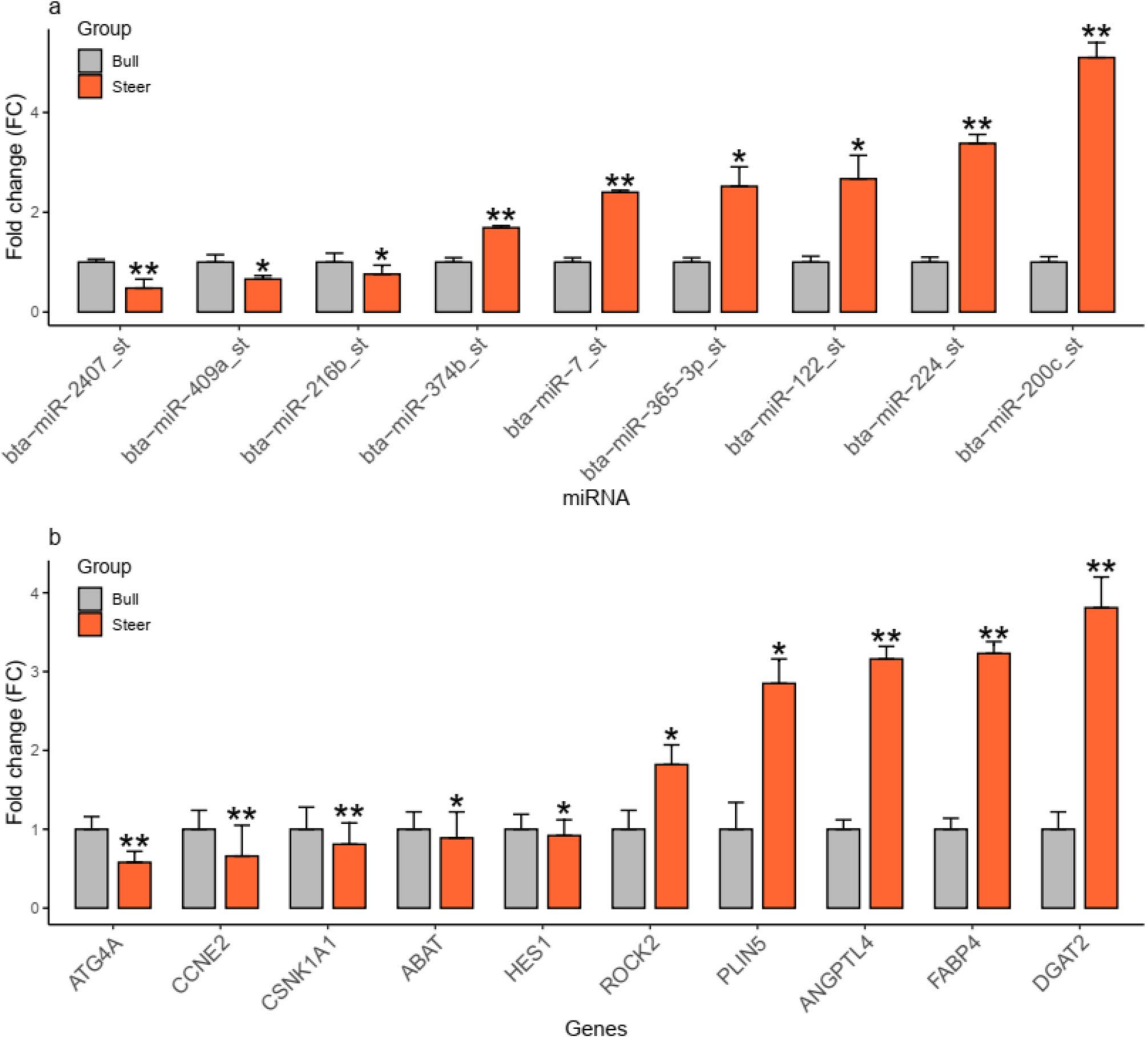
Experimental verification by using qRT-PCR. (**a**) shows the validation of differentially expressed miRNAs, *P < 0.05, **P < 0.01; (**b**) presents the validation of intersection genes, *P < 0.05, **P < 0.01.

Crude protein and crude fat were analyzed based on the semi-micro Kjeldahl method and the classic Soxhlet method. The composition analyses of fatty acid (2 g of freeze-dried sample collected from LD) were performed with a gas chromatograph (Agilent Technologies 7890A, Wilmington, DE, USA). The conditions of chromatography are as follows: column, Supelco sp-2560, 100 m × 250 μm × 0.2 μm; starting temperature, 130 °C, maintained for 3 min; after 5 min, the temperature increased to 240 °C at 4 °C/min for 50 min; and inlet temperature, 240 °C. Statistical analyses were performed with SAS 9.4. The phenotypic data of meat quality was estimated using ANOVA and expressed by mean ± standard deviation (Supplementary Tables S1 and S2). To observe genes that we predicted and cloned in advance, we preliminarily showed that the expression levels of ANGPTL4, FABP4, and DGAT2 genes in the LD are significantly higher than in bulls on predictions for previous work (15 bulls and 15 steers). The results are consistent with this study (Supplementary Table S3 and Fig. 8).

### miRNA and mRNA expression profiling arrays

miRNA and mRNA profiling using microarrays was performed on individual RNA samples (*n* = 3) isolated from the bull and steer, respectively. Sample labeling, microarray hybridization, and washing were performed based on the standard protocols of the manufacturer.

GeneChip™ miRNA 3.0 Array (Affymetrix, miRNA Profiling) was used to perform miRNA expression profiling. In brief, total RNA was tallied with Poly A and then labeled with biotin. The labeled RNAs were hybridized onto the microarray. The slides were washed and stained, and the arrays were scanned using a GeneChip Scanner 3000 TG system (Thermo Fisher Scientific, USA). The GeneChip Command Console software (version4.0, Affymetrix) was used to analyze array images for capturing raw data. Expression console (version1.3.1, Affymetrix) was used in RMA normalization. The Genespring software (version 12.5; Agilent Technologies) was used for probe filtration. DEMs were identified through fold change (FC). *P*-value (P) was calculated using the *t*-test. The threshold of significance for the differential expression set was FC ≥ 2.0 and *P* ≤ 0.05. Hierarchical clustering was performed to show the distinguishable miRNA expression pattern among samples.

GeneChip™ Bovine Genome Array (Affymetrix, Transcriptome Profiling) was applied to analyze mRNAs expression. The total RNA of mRNA samples was primarily transcribed to double-stranded cDNA, and cRNA was synthesized and labeled with biotin compared with the miRNA profiling procedure. The labeled cRNAs were hybridized onto the microarray. The processing of sample labeling, microarray hybridization, and washing was consistent with miRNA profiling.

The Affymetrix GeneChip Command Console (Version 4.0, Affymetrix) and Genespring software (Version 12.5; Agilent Technologies) were employed to capture the raw data for Probe filtration. The raw data was normalized with the MAS5 algorithm by the Genespring software (Version 12.5; Agilent Technologies). The other analysis procedures, such as FC, significant difference (P*-*value), and hierarchical clustering, were the same as miRNA profiling.

### Quantitative real-time PCR (qRT-PCR)

Part of the miRNA and intersection genes associated with fats and fatty acids were selected for verification. qRT-PCR was performed with a PCR Kit (LightCycler® 480 SYBR Green I Master, USA), and a miRNA-specific primer was used in quantifying nine relevant DEMs (Fig. 8a). U6 was selected as an internal control for the correction of analytical variations. Each primer was 10 μmol/μL. Then differentially regulated mRNAs as integration genes were used for validation (Fig. 8b). The primers used are shown in Supplementary Tables S4 and S5. The reacting system was 20 μL and used with an SYBR Premix Ex Taq™ II kit (Tli RNaseH Plus, Japan). The universal reverse primer that provided GAPDH was selected as a control for the correction of analytical variations. The final concentration of each primer was 10 μmol/μL. qRT-PCR experiments were performed in triplicate for each sample as described above. The relative expression was calculated using the 2^–ΔΔCt^ method^98^. Differences between groups were analyzed using the Student's *t*-tests for independent samples and visualized as a histogram in R (Version 3.5.3).

### Bioinformatic analysis

The miRanda algorithm was employed to predict the potential targets of all the DEMs. The transcript paired with miRNA information was extracted from the prediction results, and the annotation was obtained from the NCBI bovine (*Bos taurus*) database. Pathway, GO, and UniPort terms were analyzed using STRING 11.0 (https://string-db.org/). Intragroup co-expression analysis of mRNA was performed using the Pearson algorithm and Cytoscape (Version 3.6.1)^99^ to evaluate the co-expression genes in bulls and steers. The interaction networks of miRNA and mRNA were constructed using the Cytoscape software. MiRNAs from the most significant up- and down-regulated clusters were selected to construct the co-expression network^100^. Subsequently, the critical genes from negative correlation results in co-expression and related to significant lipid metabolism pathway were integrated and verified.

### Vector construction

The wild-type construct 3′ UTR of genes containing the targets of miRNA binding site was amplified from bovine (*Bos taurus*) genomic DNA by PCR, and the primers are shown in Supplementary Table S2. PCR products were cloned into pmirGLO Dual-Luciferase miRNA Target Expression Vector (Promega Corporation, Madison, WI, USA) by using the Nhe I and Xho I restriction sites. The mutated-type construct 3′ UTR sequences of PLIN5 share a substitution (GUGGAGUUG → CGUUUCAAC) in the binding site.

### Luciferase reporter assays

A dual-luciferase reporter assay was performed for detecting the interactions between the target genes and miRNAs. Bovine pre-adipocytes cells (BPCs) were seeded in 24-well plates at 2 × 10^4^ cells/well and cultured under routine conditions with 10% fetal bovine serum (Ausbian). When the cells reached 60%–70% confluence, pmirGLO-3′ UTR (1 µg) was co-transfected with 60 nM negative control or miRNA mimic (both from RiboBio) by using 2 μL of X-tremegene HP (Promega) according to the manufacturer's instructions. The cells with plasmid and X-tremegene HP were cultured at 37 °C in an incubator supplemented with 5% CO_2._ The relative luciferase activity was measured 24–48 h after transfection by Dual-Luciferase^®^ Reporter Assay system^101^.

#### Cell proliferation and differentiation assay

BPC model was established (Laboratory of Animal Medicine, Yanbian University, China), and cells were isolated from fatty tissues from calves. The filtered and rinsed cells were cultured at 37 °C in an incubator supplemented with 5% CO_2_. At 70% confluence, differentiation was induced with induced liquid IDI (0.5 mmol/L IBMX, 1 μmol/L DEX, and 10 μg/mL Insulin). After 48 h, the induced liquid was removed and changed to insulin-containing IDI.

#### Western blotting analysis

BPC lysed by RIPA Lysis and Extraction Buffer (ThermoFisher Technology, Beijing, China). Protein content was determined by the BCA protein assay reagent (Betotime Biotechnology, China), and 20 μg of each sample was subjected to polyacrylamide gel electrophoresis. The separated proteins were transferred to polyvinylidene fluoride (PVDF) membranes under 200 mA for 90 min. PVDF membranes were incubated with primary antibody (1:1000 dilution; PAB12542, Abnova Diagnostics, Dongguan, China), followed by incubation with horseradish peroxidase-conjugated secondary antibody (1:10,000 dilution; Zymed, San Diego, CA, USA). The membrane was re-probed with a primary antibody against GAPDH (1:3000 dilutions; Santa Cruz Biotech., Santa Cruz, CA, USA) as a control^56^. The assay was repeated to confirm the result.

### Ethical approval

All methods were carried out in accordance with ARRIVE guidelines and regulations.

## Supplementary Information

Supplementary Information 1.

Supplementary Information 2.

Supplementary Information 3.

Supplementary Information 4.

Supplementary Information 5.
