# Supplementary material for: Integrated analysis of expression profiles with meat quality traits in cattle
Source: Sci Rep. 2022 Apr 8;12:5926. doi: 10.1038/s41598-022-09998-w (PMC8993808; doi:10.1038/s41598-022-09998-w)
Supplement: Supplementary file 1 — Supplementary Information 1. [file 41598_2022_9998_MOESM1_ESM.docx]

**Integrated analysis of expression profiles with** **meat quality traits in cattle**

**Authors**

Yunxiao Li^1^, Miaosen Yang^5^, Angang Lou^3^, Jinyan Yun^8^, Chunyu Ren^9^, Xiangchun Li^3^, Guangjun Xia^3^, Kichang Nam^4^, Duhak Yoon^6^, Haiguo Jin^7^, Kangseok Seo^4^*, Xin Jin^2^*

**Affiliations**

^1^College of Life Science, Shandong University, Qingdao, China;

^2^Engineering Research Center of North-East Cold Region Beef Cattle Science & Technology Innovation, Ministry of Education, Yanbian University, Yanji, China;

^3^Department of Veterinary Medicine, College of Agriculture, Yanbian University, Yanji, China;

^4^College of Life Science and Natural Resources, Sunchon National University, Sunchon, South Korea;

^5^Department of Chemistry, Northeast Electric Power University, Jilin, China;

^6^Department of Animal Science, Kyungpook National University, Taegu, South Korea;

^7^Branch of Animal Husbandry, Jilin Academy of Agricultural Sciences, Changchun, China;

^8^College of Animal Science and Technology, Jilin Agricultural Science and Technology University, Jilin, China;

^9^Animal Husbandry Bureau of Yanbian Autonomous Prefecture, Yanji, China

*Co-corresponding author:

Kangseok Seo: sks@sunchon.ac.kr

Xin Jin: jinxin@ybu.edu.cn

**Supplementary Information**

**Supplementary Figure S1.** Volcano plot for differentially expressed miRNAs and mRNA.

**Supplementary Figure S2.** Hierarchical clustering for significant miRNA.

**Supplementary Figure S3.** Hierarchical clustering for significant mRNA.

**Supplementary Figure S4.** The expression trend in the cluster of miRNA and mRNA.

**Supplementary Figure S5.** Congruent relationship of intersection genes with Significantly enriched GO, Pathway, and UniProt.

**Supplementary Figure S6**. The corresponding relationship of 26 intersection genes with the miRNA-mRNA interaction network.

**Supplementary Figure S7.** Hub genes of the top 10 based on intersection gene set.

**Supplementary Figure S8.** Full-length blots in parallel experiments.

**Supplementary Table S1.** Phenotypic differences related to fat between bulls and steers.

**Supplementary Table S2.** Phenotypic differences related to fatty acid between bulls and steers.

**Supplementary Table S3.** Expression differences of predicted genes related to fat deposition between bulls and steers (15 bulls and 15 steers)

**Supplementary Table S4.** miRNA-specific primers for qRT-PCR.

**Supplementary Table S5.** qRT-PCR primers for integration genes.

**Supplementary Table S10.** Feed composition during the experiment.


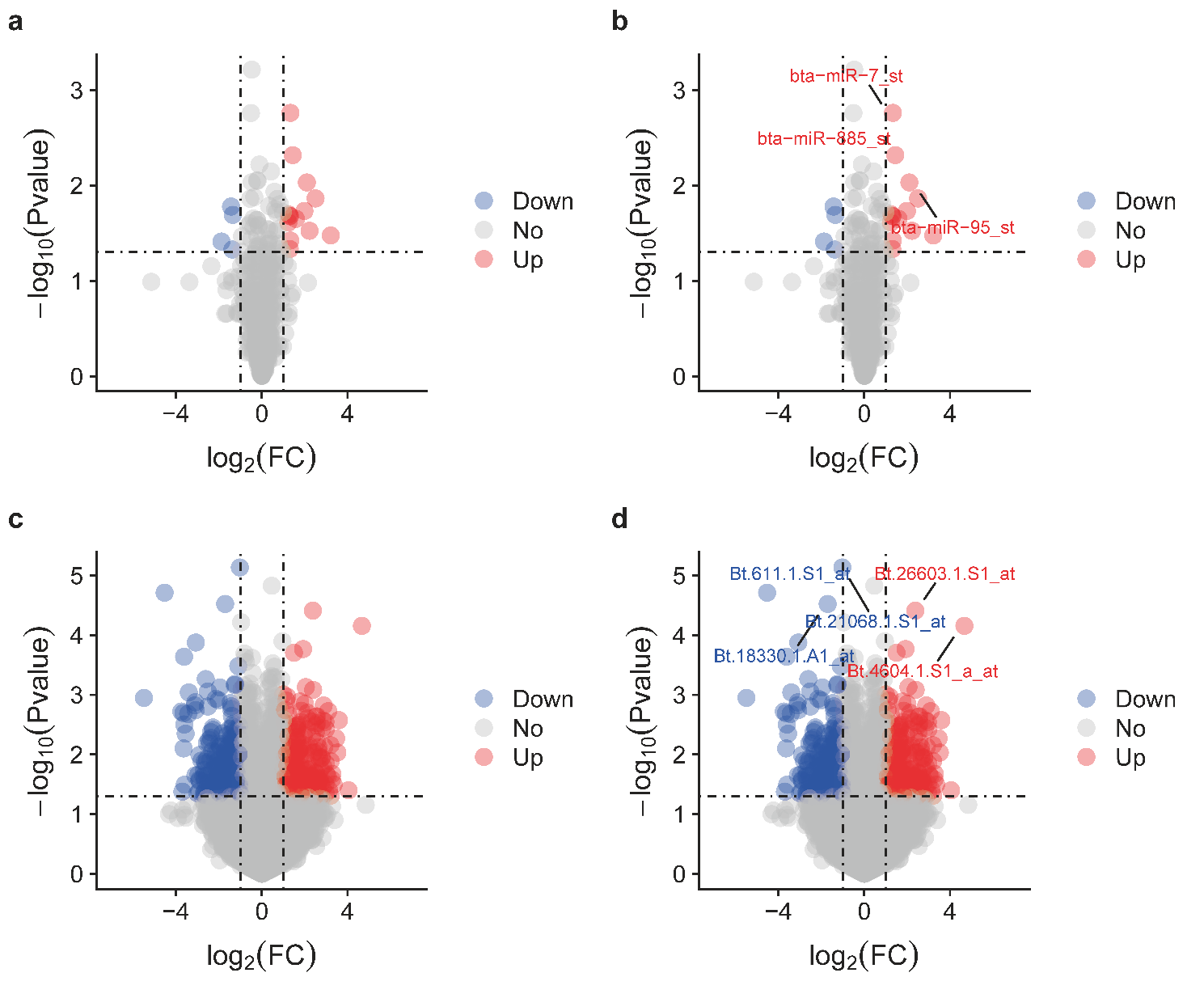


**Supplementary Figure S1.** Volcano plot for differentially expressed miRNAs and mRNA. Supplementary Figure S1a and Figure S1b described all expression of miRNAs and significant miRNAs (Red and blue indicate up- and down-regulation, respectively; grey indicate non-significant) with labels (P<0.01). Figure S1c and Figure S1d described all expressions of mRNAs and significant miRNAs (Red and blue up- and down-regulation, respectively; grey representing non-significant) with labels (P<0.0001).


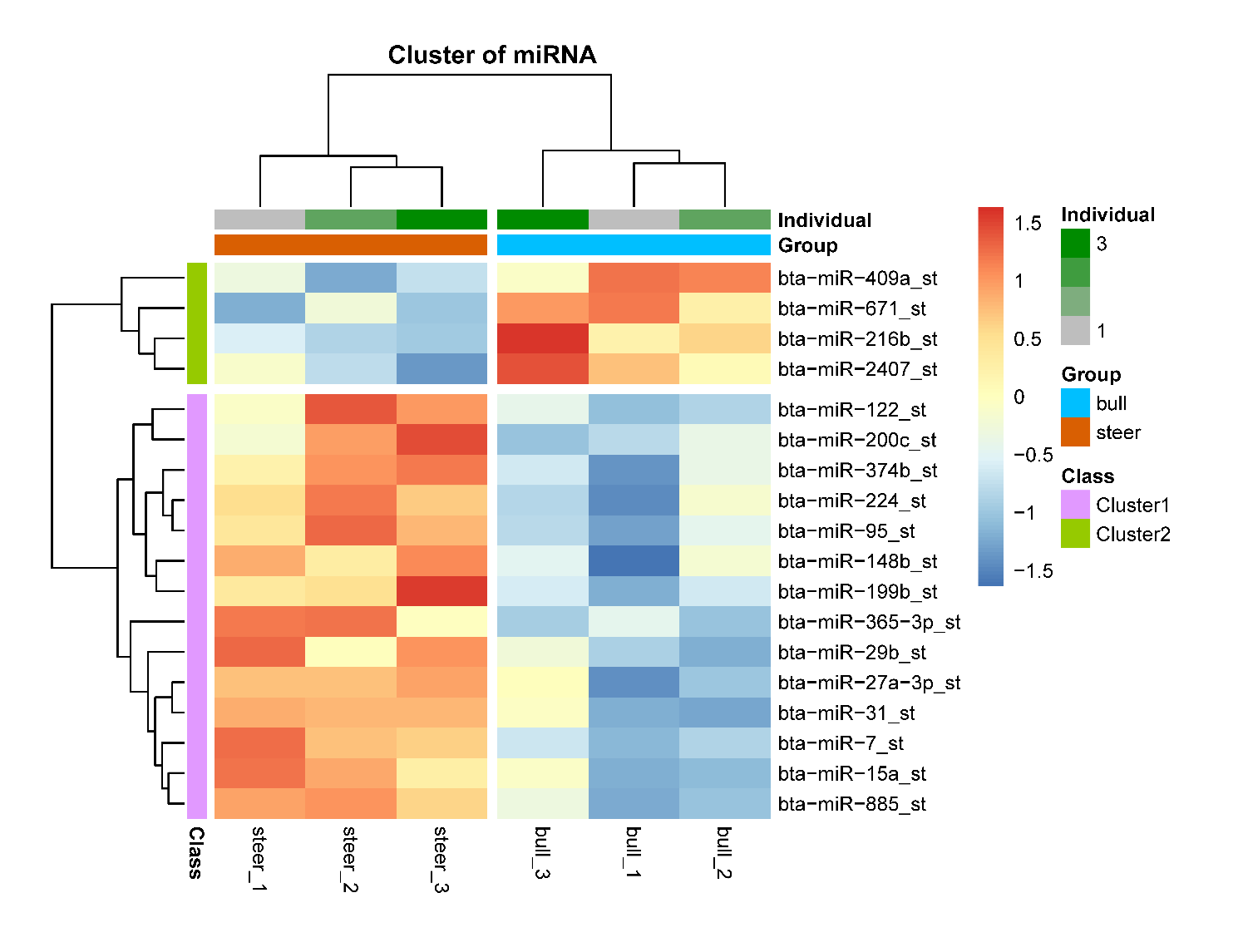


**Supplementary Figure S2.** Hierarchical clustering for significant miRNA. Significantly expressed miRNAs are separated into two clusters in bulls and steers (Orange and blue indicate high-and low-expression abundance, respectively).


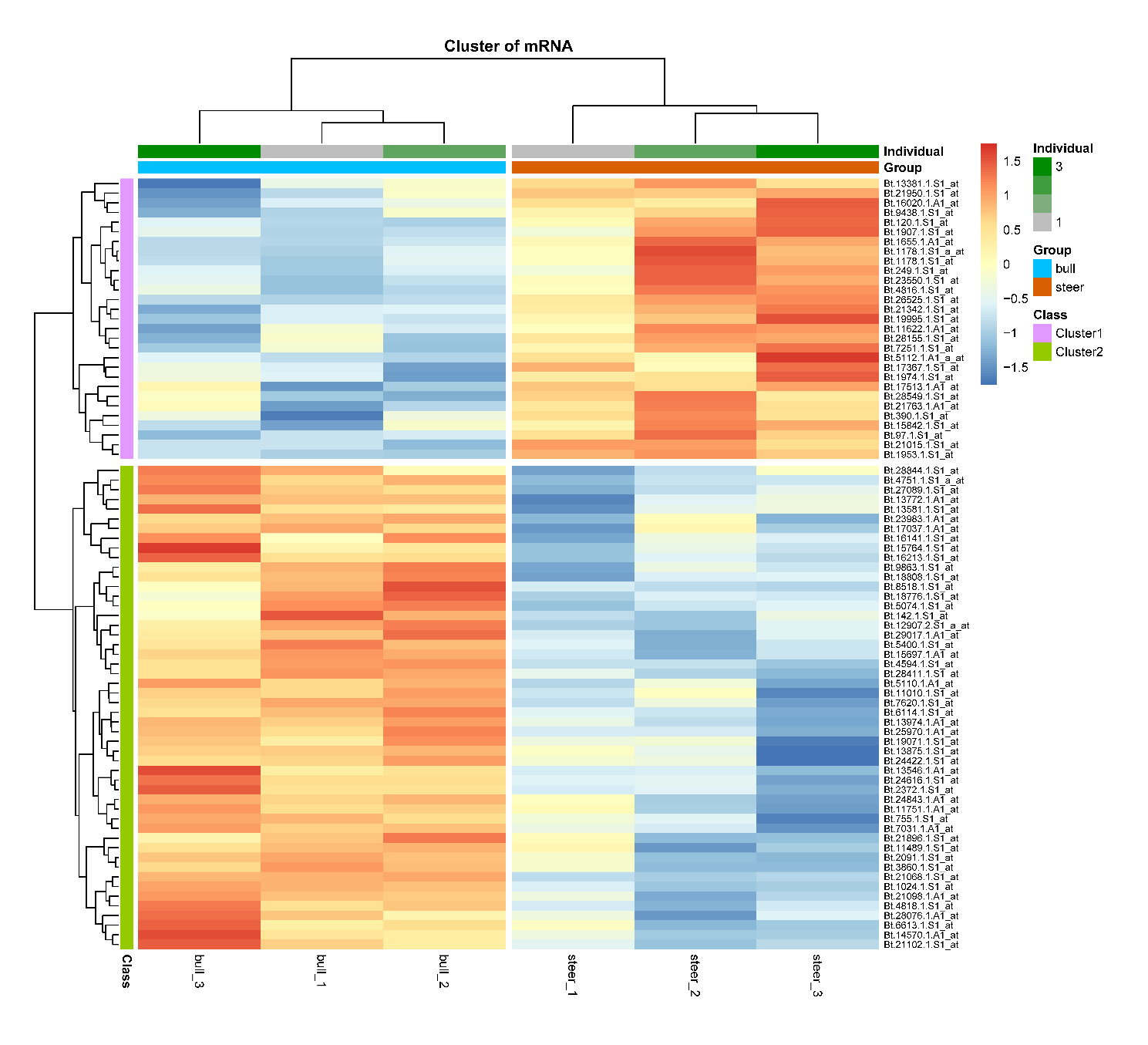


**Supplementary Figure S3.** Hierarchical clustering for significant mRNA. Significantly expressed mRNAs are separated into two clusters in bulls and steers (Orange and blue indicate high-and low-expression abundance, respectively).


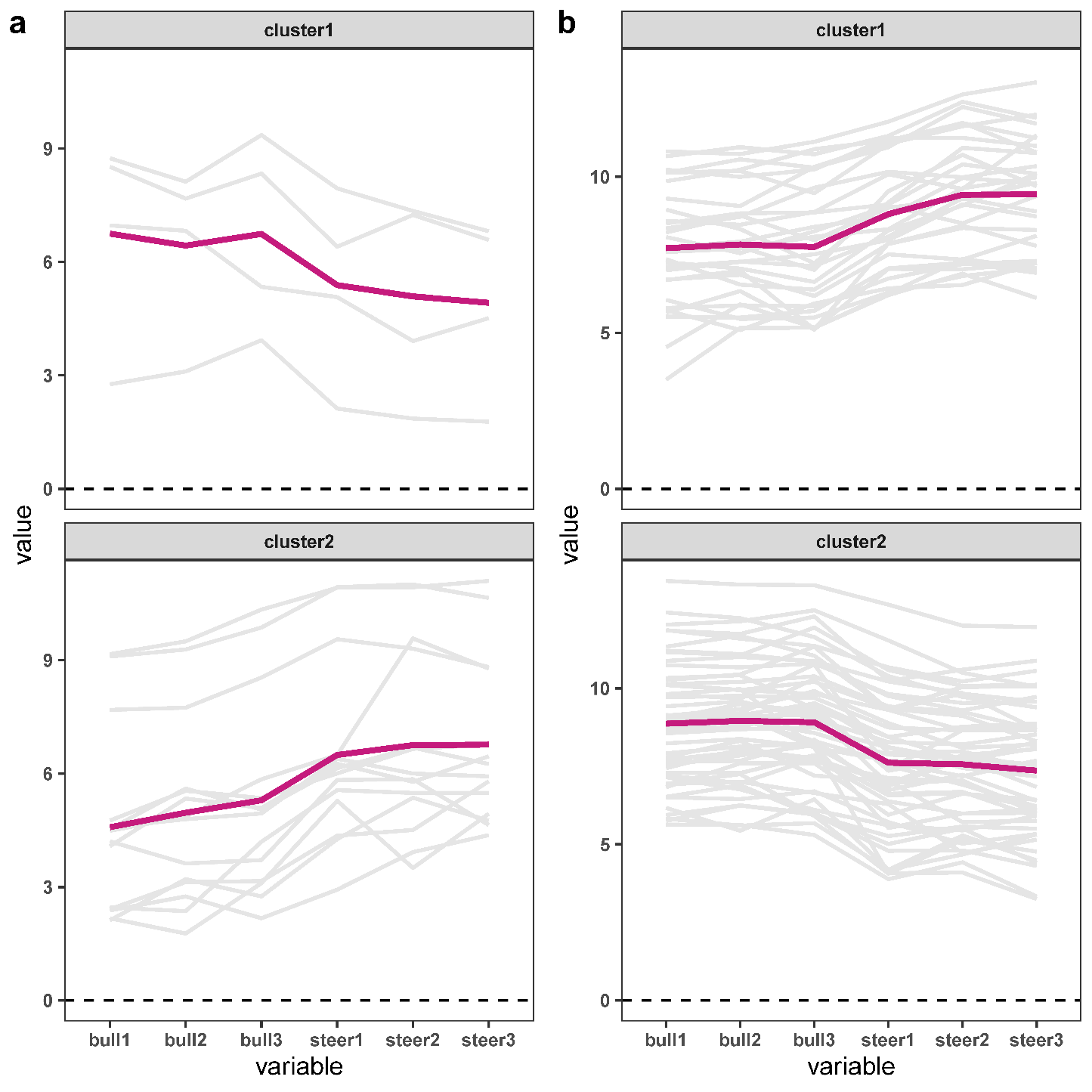


**Supplementary Figure S4.** The expression trend in the cluster of significant miRNA (Fig. S4a) and mRNA (Fig. S4b).


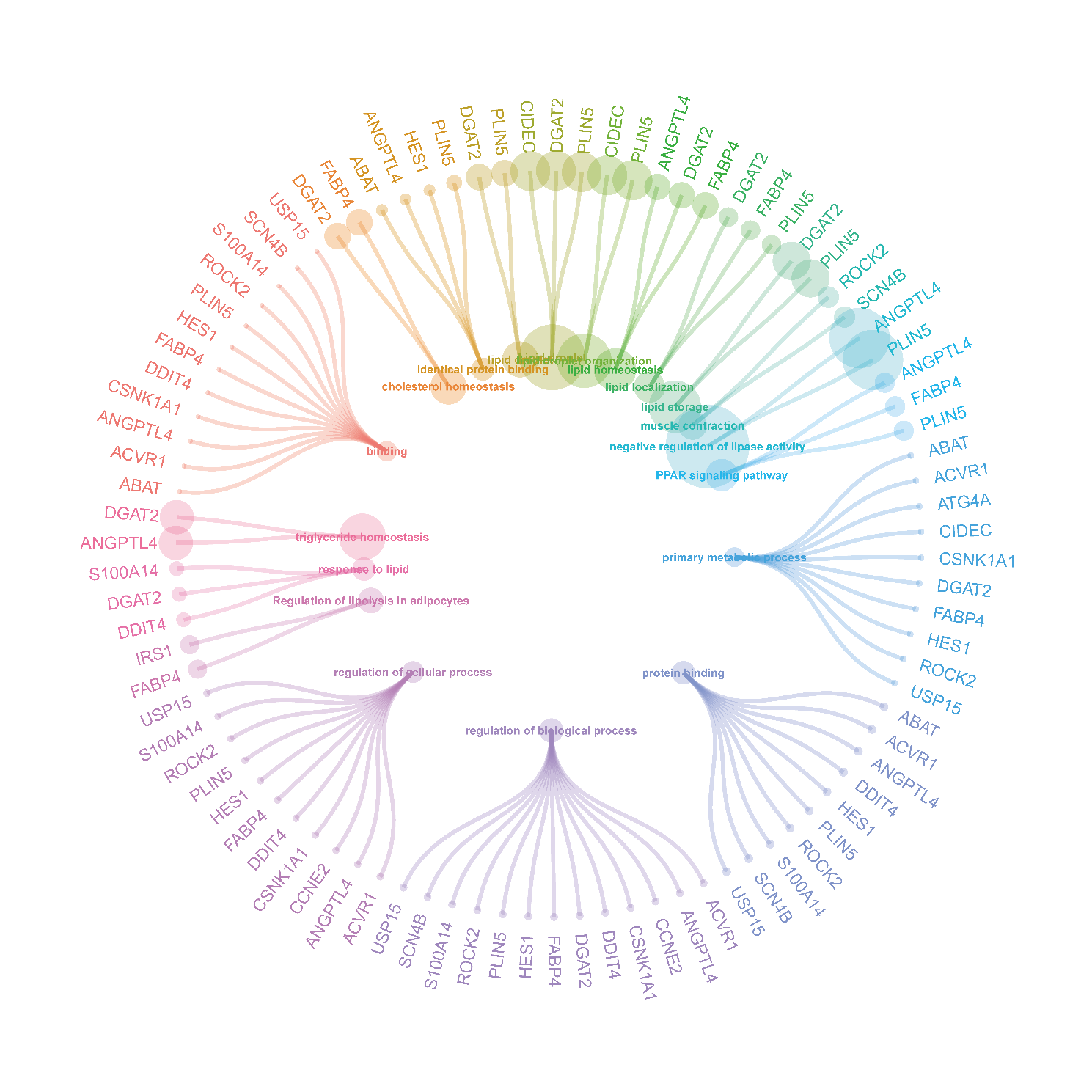


**Supplementary Figure S5.** Congruent relationship of intersection genes in Significantly enriched GO, pathway, and UniProt. P≤0.05.


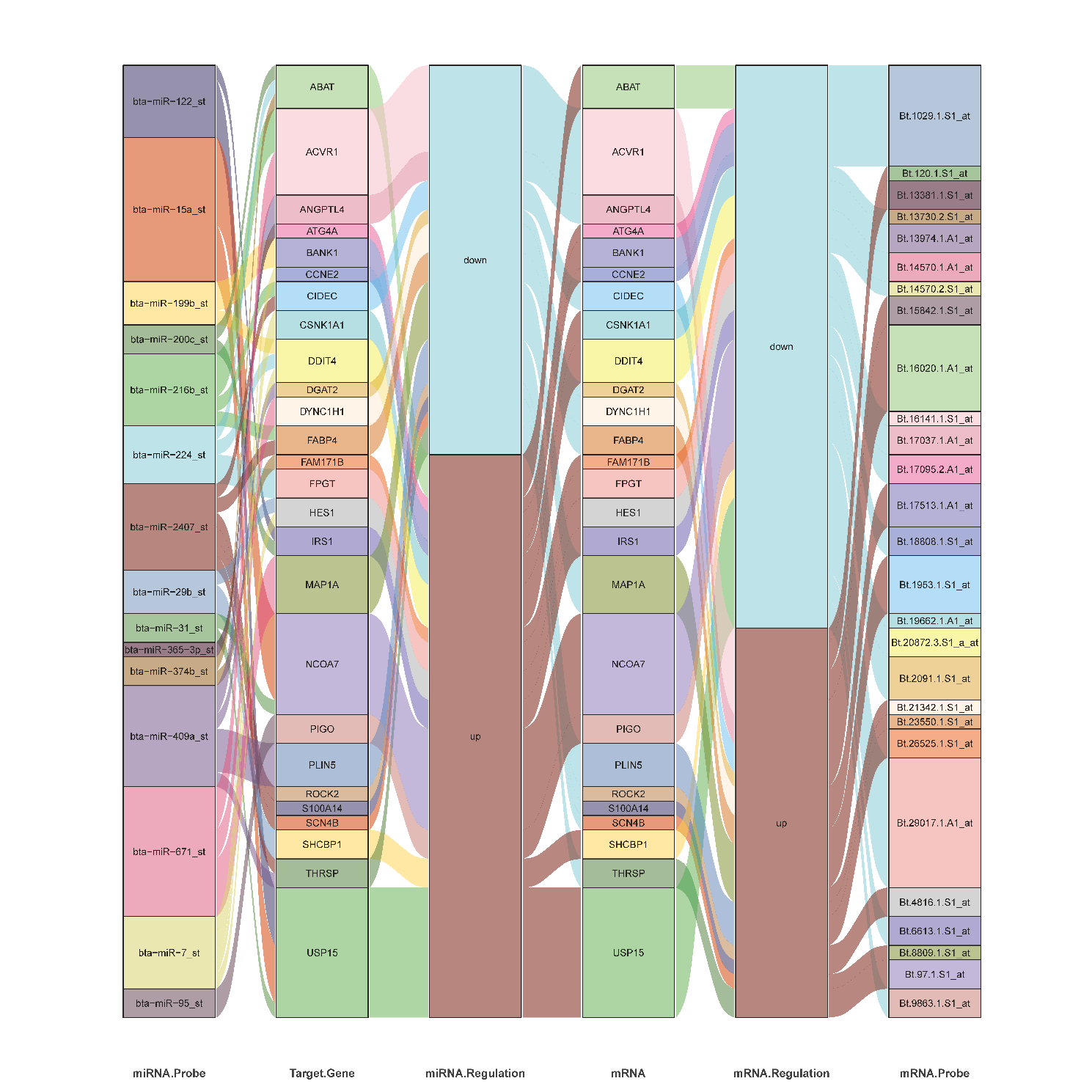


**Supplementary Figure S6.** The corresponding relationship of 26 intersection genes in the miRNA-mRNA interaction network.


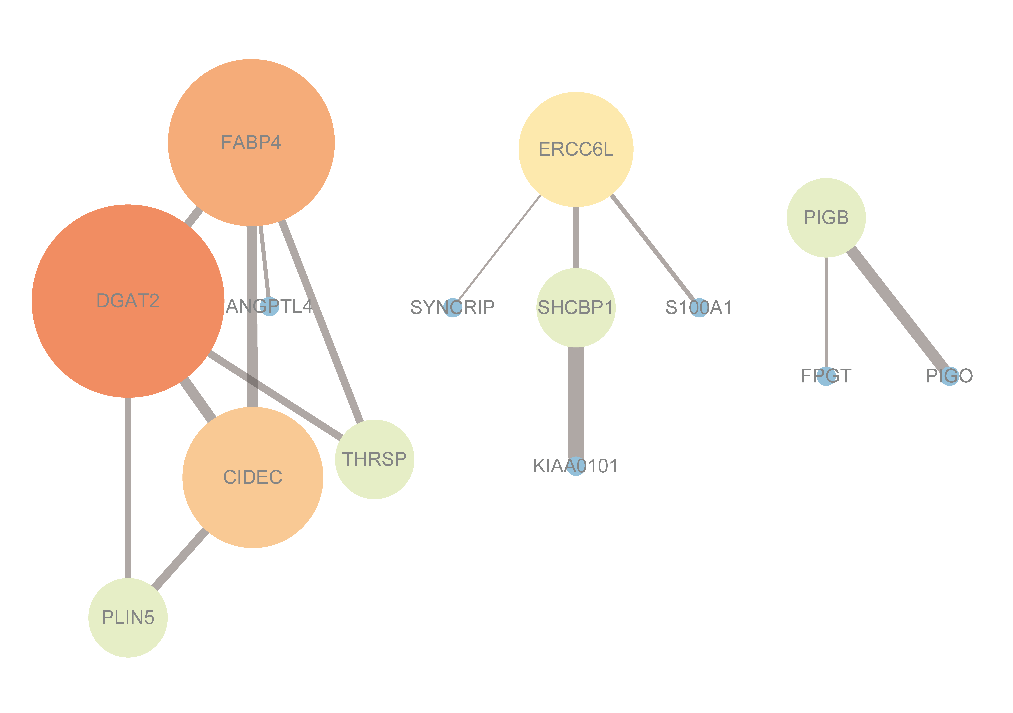


**Supplementary Figure S7.** Hub genes of the top 10 based on intersection gene set. Multiple algorithms are performed to estimate hub genes network; Hub genes are ranked by color and classed three clusters (MCC algorithms); There are three kinds of colors in the figure, with orange representing the most strong centrality, followed by green and blue.


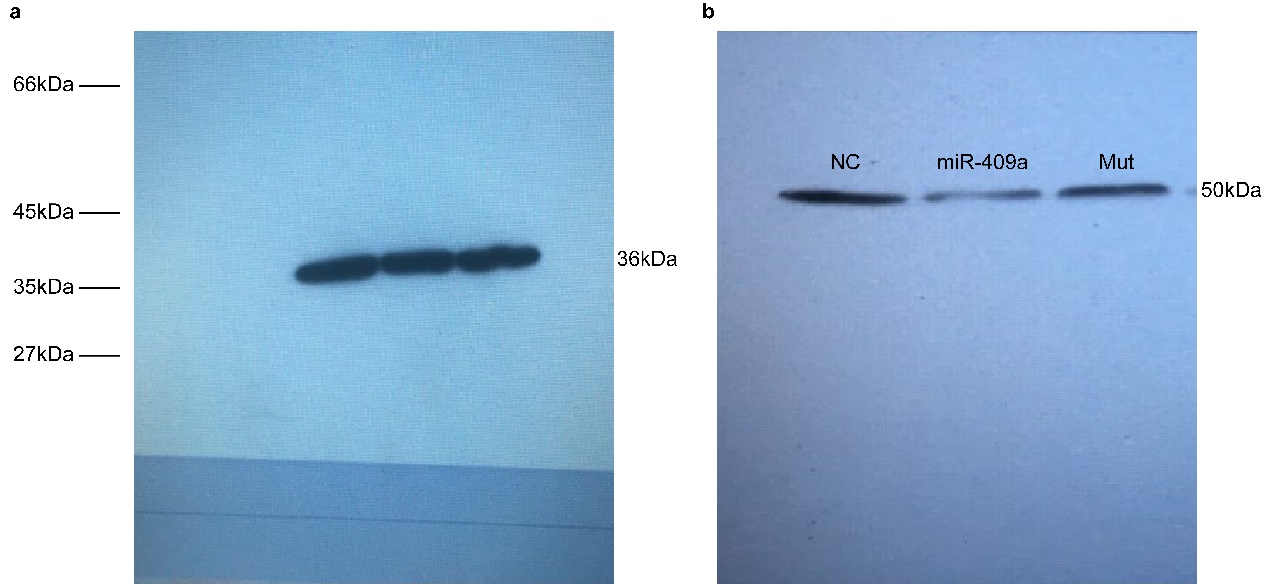


**Supplementary Figure S8.** The blots in parallel experiments. Supplementary Figure S8a and Figure S8b are GAPDH (36kDa) and blot of PLIN5 (50kDa), respectively. In Supplementary Figure S8b, three tracks were captured from the original full-length membrane image that included multiple lanes of PLIN5, which presented in the same order consistent with Figure 7a.

| **Traits** | **Crude fat (%)** | **Crude protein (%)** |
| --- | --- | --- |
| Bulls | 13.26 ± 6.45^a^ | 26.47±2.15^a^ |
| Steers | 21.43 ± 6.89^b^ | 22.22±4.07^b^ |

**Supplementary Table S1.** The differences of fat between-group bulls and steers. Lowercase and uppercase superscripts in a row indicate a significant difference (P<0.05).

| **Fatty acid** | **Fatty acid contents (mg/g)** | | **Fatty acid composition (%)** | |
| --- | --- | --- | --- | --- |
|  | **Bulls** | **Steers** | **Bulls** | **Steers** |
| Lauric acid (C12:0) | 0.06 ± 0.05^A^ | 0.23 ± 0.07^B^ | 0.05 ± 0.01^A^ | 0.08 ± 0.01^B^ |
| Myristic acid (C14:0**)** | 3.06 ± 2.71 ^A^ | 10.26 ± 3.18 ^B^ | 2.46 ± 0.51^A^ | 3.53 ± 0.47^B^ |
| Myristtoleic acid (C14:1**)** | 0.42 ± 0.33^A^ | 2.73 ± 1.33^B^ | 0.39 ± 0.19^A^ | 0.93 ± 0.37^B^ |
| Palmitic acid (C16:0) | 31.12 ± 23.55 ^A^ | 85.93 ± 23.49 ^B^ | 26.54 ± 2.25^A^ | 29.64 ± 1.65^B^ |
| Palmitoleic acid (C16:1) | 4.90 ± 4.60 ^A^ | 17.35 ± 6.59 ^B^ | 3.95 ± 0.83^A^ | 5.85 ± 0.81^B^ |
| Stearic acid (C18:0) | 17.67 ± 7.61 ^A^ | 32.74 ± 6.85 ^B^ | 17.48 ± 3.39^A^ | 11.52 ± 1.20^B^ |
| Oleic acid (C18:1) | 49.40 ± 38.69 ^A^ | 130.07 ± 32.75 ^B^ | 41.98 ± 3.28^A^ | 45.24 ± 1.74^B^ |
| Linoleic acid (C18:2) | 5.77 ± 0.79 ^A^ | 8.19 ± 1.66 ^B^ | 6.72 ± 3.28^A^ | 2.91 ± 0.49^B^ |
| Linolenic acid (C18:3) | 0.45 ± 0.24 ^A^ | 0.89 ± 0.22 ^B^ | 0.44 ± 0.15^A^ | 0.31 ± 0.04^B^ |
| Saturated fatty acid (SFA) | 51.91 ± 33.32 ^A^ | 129.16 ± 32.92 ^B^ | 46.52 ± 1.92 | 44.77 ± 1.49 |
| Unsaturated fatty acid (UFA) | 60.94 ± 44.25 ^A^ | 159.23 ± 41.54 ^B^ | 53.48 ± 1.92 | 55.23 ± 1.49 |
| Monounsaturated fatty acid (MUFA) | 54.72 ± 43.36 ^A^ | 150.15 ± 40.11 ^B^ | 46.32 ± 3.94^A^ | 52.01 ± 1.51^B^ |
| [Polyunsaturated](javascript:;) [fatty](javascript:;) [acids](javascript:;) (PUFA) | 6.22 ± 1.02 ^A^ | 9.09 ± 1.87 ^B^ | 7.16 ± 3.36^A^ | 3.22 ± 0.53^B^ |
| Total fatty acids (TFA) | 112.85 ± 77.31^A^ | 288.40 ± 73.87 ^B^ | 100 | 100 |

**Supplementary Table S2.** Phenotypic differences related to fatty acid between bulls and steers. Uppercase superscripts in a row indicate an extremely significant difference (P<0.01).

| **Group** | **Genes expression** | | |
| --- | --- | --- | --- |
|  | **ANGPTL4** | **DGAT2** | **FABP4** |
| Bulls | 7.97 ± 0.97^a^ | 4.43 ± 1.15^a^ | 8.92 ± 0.61^A^ |
| Steers | 10.51 ± 0.64^b^ | 7.45 ± 0.55^b^ | 46.36 ± 3.54^B^ |

**Supplementary Table S3.** Expression differences of predicted genes related to fat deposition between bulls and steers (15 bulls and 15 steers). Lowercase and uppercase superscripts in a row indicate a significant difference (P<0.05) and an extremely significant difference (P<0.01), respectively.

| **miRNA** | **Prime** | **TM(℃)** |
| --- | --- | --- |
| bta-miR-122 | TGGAGTGTGACAATGGTGTTTG | 60 |
| bta-miR-216b | AAATCTCTGCAGGCAAATGTGA | 60 |
| bta-miR-200c | TAATACTGCCGGGTAATGATGGA | 60 |
| bta-miR-224 | CAAGTCACTAGTGGTTCCGTTTA | 60 |
| bta-miR-2407 | CTGGGCGGATGGGAAGGGCTGG | 60 |
| bta-miR-409a | AGGTTACCCGAGCAACTTTGCAT | 60 |
| bta-miR-7 | TGGAAGACTAGTGATTTTGTTGTT | 60 |
| bta-miR-374b | ATATAATACAACCTGCTAAGTG | 60 |
| bta-miR-365-3p | TAATGCCCCTAAAAATCCTTAT | 60 |
| U6^§^ | CAAGGATGACACGCAAATTCG | 60 |

**Supplementary Table S4.** miRNA-specific primers for qRT-PCR. ^§^QIAGEN kit.

| **Gene** | **Accession ID** | **Sequence (5’ to 3’)** | **Size (bp)** |
| --- | --- | --- | --- |
| FABP4 | NM_174314.2 | F-GGATGACGTTTGCATCGAACT  R-CCATATCAAAATCAGTCTGGAAGAA | 117 |
| ATG4A | NM_001001171.1 | F-GCCACAGTCTTTAGGGGCAT  R-TGGAAAGTCTGGTCGTCAGC | 153 |
| ANGPTL4 | NM_001046043.2 | F-CTGCAGGAAATGCCCTCTGA  R-TCCTCCAGCCAGTCAATGTG | 196 |
| ABAT | NM_001081581.1 | F-TCGCAGACTTACCCGCATAC  R-TGGGCATAGGGTCATCCGTT | 111 |
| DGAT2 | NM_205793.2 | F-AGCATCATGGGTGTCTGTGG  R-TAAAGGCCCAGATGTGGCAG | 161 |
| ROCK2 | NM_174452.2 | F-GGTTTTCTGGGCTCCTTTGG  R-CTGGCAAATGTGCTGGTGAA | 202 |
| PLIN5 | NM_001101136.1 | F-TAGCCGAAGCCTTGGTCTTG  R-ACAGCCAGTGAAACCAGCTT | 125 |
| IRS1 | XM_003585773.3 | F-CTGGAGTCCGTGTGAATGCT  R-CAGGGAGCTCAACCCTTCAG | 90 |
| CCNE2 | NM_001015665.1 | F-GGAGGGACAGTTGTCACCAG  R-GGGAATTGGCAGGACAGTCA | 196 |
| CSNK1A1 | XM_005209556.2 | F-CCACAGGCAAGCAAACTGAC  R-GGACACAGCATCCATCACCA | 235 |
| HES1 | NM_001034678.1 | F-GTTTGCCTTCCTCATCCCCA  R-GTCTCCACATGGAGTCTGCC | 146 |
| CIDEC | XM_010817501.3 | F-CCTCAGCTCTAGGGGACCAT  R-CCATTGCCTGCTGGTGACTA | 156 |
| THRSP | NM_001040533.1 | F-CGCCTCCGATCTCTACAACT  R-CTAGCGAAGTGCAGGTGGAA | 213 |
| GAPDH | BC102589 | F-ACCCAGAAGACTGTGGATGG  R-ACGCCTGCTTCACCACCTTC | 247 |

**Supplementary Table S5.** qRT-PCR primers for genes.

| Feed composition  (%) | Bodyweight (kg) | | | | |
| --- | --- | --- | --- | --- | --- |
|  | 130~230 | 230~330 | 330~500 | 500~600 | 600~700 |
| Corn | 66 | 69 | 75.5 | 82.5 | 86 |
| Bran | 5 | 5 | 3.5 | 3.5 | 3 |
| Soybean | 25 | 22 | 17 | 10 | 7 |
| Premixe | 4 | 4 | 4 | 4 | 4 |

**Supplementary Table S10.** Feed composition during the experiment.
